# A Treatise upon Cerebral Pathology or the Diseases of the Brain

**Published:** 1844-10-01

**Authors:** 


### Traité de Pathologie Cerebrale ou des Maladies du Cerveau. Nouvelles Recherches sur sa Structure, ses Fonctions, ses Alterations, et sur leur Traitement Therapeutique, Moral et Hygienique. Par *Scipio Pinel.* Paris, 1844. 8vo. pp. 560.

A Treatise upon Cerebral Pathology or the Diseases of the Brain. By *Scipio Pinel.*

This work, principally devoted to the consideration of the changes which occur in the brain in the various forms of insanity, is intended by its author to be of an elementary and practical character, presenting to the student and practitioner an abridged but faithful summary of all that is known respecting the structure, functions, and diseases of the brain. It embraces several of his own researches, which he regards as new, upon acute inflammation of the brain, the indurated and œdematous condition of that organ, and upon general paralysis. He has divided the book into nine Chapters; the first treating of the General Pathology of the Nervous System, the second and third, of the Anatomy and Physiology of the Brain; the four next describe the various lesions, and the two last, enumerate their causes and detail their treatment. It is intended to give some notice of each of these Sections.

### Chap. I.—General Principles of the Pathology of the Nervous System.

The author, after enumerating the various classifications of mental diseases adopted by Pinel, sen., Foderé, Georget, Esquirol, &c. observes that in all these the disorders of the understanding are taken as the basis, while the lesions of the various other functions of the nervous system are treated of as quite secondary, or are not even mentioned at all. In the brain, three principal functions are intimately combined, viz. the understanding, motility, and sensibility; and, although these possess very different characteristics, and are referrible to different localities, yet their relations in structure and function are so intimate, that they cannot be separated from each other, either in the study of the healthy or diseased conditions of the economy.

"If for a moment you can take courage to forget all that has been written upon this subject, and if as an impartial observer you enter amidst those vast assemblages of nervous affections which present themselves under every form and in every stage in the two great hospitals devoted to them at Paris, what do you first remark upon taking a general view of them. You there see some in a state of furious delirium, a greater number in calmer delirium, many more still in a state of simple dementia or paralytic dementia: you see entire sections of imbeciles, idiots, epileptics, and hysterico-epileptics; who constantly manifest extraordinary lesions of certain faculties, of certain desires, of the most grave disorders of the senses, of general sensibility, of motility and contractility. If you had the opportunity of observing these patients from the period of their admission to their death, and following them through their successive degradations, you would observe them for the most part pass insensibly from the state of furor to that of mild delirium, then to the condition of dementia, paralysis, convulsions, epilepsy, imbecility, and so on, to the annihilation of every physical and moral power. Now is it not allowable for one who has had such a spectacle before his eyes for twenty years, to take a general view of this subject, and to believe that all these successive disorders, even while they constitute distinct affections, are in reality but more or less prolonged periods of the same disease. They are the expressions, in fact, of a series of alterations in the brain, which in the acute stage exalt at first all the sensory and motory powers of the organ, as in furor, hallucinations, acute delirium, and mania; which latter work a gradual change in its properties, and slowly give rise to the loss of memory, confusion and obliteration of ideas; and which, penetrating amid the motor and sensory fibres of the interior of the brain, then produce all the symptoms of paralysis, contractions, and insensibility; until, at last, affecting the most sensitive and most irritable fibres, such as those of the optic thalami, the peduncles, pons varolii, and medulla oblongata, produce the convulsive and epileptic attacks that presage the fatal termination of this slow succession of disorganizing processes which the cerebral mass has been undergoing. We are well aware that the affection of the brain does not always follow the regular progress; and that it may be first manifested either by epilepsy, or more rarely by paralysis, or by some other lesion of the understanding, motility, or sensibility. It little matters whether it rises from the base towards the periphery, or descends from the surface to the base. This progress does not influence the general results, and the rapid sketch we have drawn is sufficient to show the connexion and succession of all these cerebral affections, which with so little reason have been forcibly separated from each other."

Upon the foregoing principles Dr. Pinel founds his classification of affections of the brain, adopting indeed that which had been already in part developed by Andral. All changes in the brain, whether slight or extensive, give rise to certain derangements of functions, which are observed, either alone or united, under the following orders. 1. Lesions of the Understanding. 2. Lesions of the Propensities and Instincts. 3. Lesions of the External Senses and of Sensibility. 4. Lesions of Voluntary Motion.

The author protests strongly against the practice of regarding mental diseases as distinct in their nature from other affections of the brain. He regards it just as rational as it would be on the part of a pathologist, who, treating of the diseases of the respiratory organs, should confine himself to their most frequent symptom, the cough, should divide and subdivide this according to its various kinds, and having thus converted it into a special branch of medicine, should maintain that there existed no connection between the cough and the various bronchial and pulmonic changes. This is, however, done daily with regard to disorders of the intellect without its absurdity being perceived. Diseases of the brain, whether slight or severe, should be subjected to the same principles which regulate ordinary pathology, and the first step towards the accomplishment of this, is to remove the sense of vagueness with which nervous diseases are regarded. Every nervous symptom depends upon a temporary or permanent lesion, an organic change, whether slight or serious, in the structure of the nerves or in the nervous centres; and, if the morbid condition continues for some time, an alteration of structure appreciable by our senses, the microscope, or by chemical analysis, is always to be found. Those who fail to discover this often do so from their imperfect acquaintance with the normal structure. A long experience in making these examinations can alone effectually show the almost constant correspondence between the functional lesions and the organic changes. Such changes are always found, but their form and appearances are often very variable, and quite peculiar to the nervous system, and of which the language in ordinary use forms a very imperfect means of description.

### Chap. II.—Anatomy of the Brain.

This Chapter contains an account of the comparative anatomy of the brain, a full description of its healthy condition in man, and a general view of the morbid changes observed in it by various writers on insanity. An analysis of what is itself but a summary would be scarcely intelligible, and we will not attempt it.

*Morbid Changes in the Brain in Insanity.*—Of 216 cases reported by Greding, the bones of the cranium were found much thickened in 167, and very thin in 38. The most frequent and most manifest alterations were situated in the membranes of the brain. Soemmering attributes mania to inflammation of the brain. The observations of Haslam are fertile in examples of morbid changes after death of various kinds situated both in the membranes and the substance of the brain. Portal prefers doubting the sufficiency of his own senses to believing that a mental malady can exist without physical changes in the brain. Esquirol, writing in 1812, described various morbid changes, especially in the membranes of the brain, and accompanied by effusions; but, writing in 1835, he states that post-mortem examinations are insufficient in determining the material causes of insanity. A strange contradiction, considering the great progress these investigations had made in this interval. Gall and Spurzheim admit the frequency of sufficient causes of insanity being discoverable after death. M. Lallemand attributes very opposite lesions to one cause —meningitis. M. Foville states, as the result of his numerous researches, that the brains of the insane present, in general, the anatomical appearances seen in inflammation, and that such are seen more often in the brain than in its membranes. Changes in the cortical substance are the most constant of all, and are directly connected with the derangements of the understanding; while those occurring in the white portions influence the movements. MM. Bouchet and Cazanvieilly conclude that epilepsy is a chronic inflammation of the white substance, and that mental alienation arises from inflammation of the cortical portion. M. Calmeil attributes the paralysis of the insane to inflammation of the grey substance and of the membranes of the brain. The same changes have been observed by Davidson in more than 200 insane patients. Bertolini observed morbid changes, especially in furious mania and in dementia with paralysis. M. Ferrus declares that, according to his experience, the alterations produced by nervous diseases, are as distinct as those occurring in any other part of the body. M. Parchappe reports a great variety of morbid appearances occurring in 130 bodies examined by him during three years. The result of the whole, according to our author is, that there are superabundant facts for constituting a correct pathological anatomy of the brain as of that of any other organ; and that even the multiplicity of facts gives rise to a degree of confusion, which he hopes, by bringing each malady in approximation with its appropriate organic change, to be instrumental in removing in the present work.

### CHAP. III.—PHYSIOLOGY OF THE BRAIN.

This chapter contains a tolerably good but condensed resumé of the more modern researches upon the physiology of the cerebro-spinal system. It is however not a little surprising that a treatise, professing to afford the latest information upon this subject, and especially with a view to the treatment of the diseases of the nervous system, should not contain the most distant allusion to the excito-motory theory, as developed by Hall, Müller, and other distinguished observers.

Although the author is a strong advocate for the localization of the various cerebral actions, he is no admirer of the organology of Gall; and takes several opportunities in the course of his work to criticise it.

### CHAP. IV.—LESIONS OF THE UNDERSTANDING.

These are manifested under three forms very distinct from each other. 1. In a state of *exaltation,* comprising acute delirium; mania or maniacal furor; monomania of ideas separable from monomania of the propensities; and cerebral hallucinations separable from illusions of the senses. 2. In a state of *feebleness,* comprising chronic delirium, (melancholia, lypemania); stupor, or cerebral œdema; and ordinary dementia. 3. In a state of *abolition,* more or less complete, comprising idiotcy and imbecility.

#### 1. *In a State of Exaltation.*

A. *Acute Delirium.*—This differs from other disorders of the intellectual faculties by its shorter duration, during which period, however, it may present the same symptoms as they do. It is accompanied by much febrile action, and is usually caused by some phlegmasia. Dupuytren has exactly described it, when sympathetic of local injury, under the name of nervous delirium. Few diseases, whether acute or chronic, terminate fatally without this occurring. It is produced by alcohol, as seen in drunkenness, and narcotic substances. Derivatives are useful; but general bleeding often leads to a condition of chronic mania.

B. *Acute Mania or Maniacal Furor* (*Acute Cerebritis.*)—Disease is frequently making insidious progress before the attack of mania excites alarm. Peculiarity and change of manners and habits may often be remarked even for months prior. The invalid sometimes makes great resistance to the progress of his infirmity, and endeavours to conceal it. Pains and heat of the head, feverishness and restlessness, with derangement of the digestive or uterine functions, are common. Confirmed delirium becomes in some gradually established, and in others more suddenly so. At the commencement, the disorder of the intellectual functions predominates, and when the disease has once declared itself, its symptoms are marked, and not to be mistaken. The period of excessive excitement varies from some days to many months or years; but, in the great majority of cases, some remission of symptoms is found to occur about the second or third month. Such diminution of symptoms is, under proper management, usually the prelude towards a cure, which is sometimes operated in a very short time, but usually not until after six months, a year, or longer.

Acute mania is terminated either by a cure, a passage into a chronic incurable state, or in many cases by death, which is often very sudden. The cure is sometimes as sudden as the invasion, but this is rare, and is in such case seldom permanent. One of the most certain signs of convalescence is a return of the natural affections for friends and relations. The calm and tranquil condition of the countenance is much to be depended upon, except in the case of monomaniacs. As a cure approaches, the face often becomes thin, and the eyes sunken. This does not arise from actual loss of substance, but from the diminution of the tension and erethism of the parts; the blood, in place of being carried towards the head, remaining in the large vessels and splanchnic cavities. Although the doctrine of crises was carried too far by the ancients, there can be no doubt that critical discharges and excretions frequently do occur, and patients who have experienced such are less liable to relapse.

Sudden death during the stage of violent excitement, which may have perhaps lasted from two or three to thirty days, is by no means rare. One is surprised to find a patient dead in the morning, who, the evening before, was in a condition of excessive excitement. At other times, after months or years of agitation, a sudden calm and silence succeeds, and in a few days the patient dies in a state of complete stupor and paralysis.

During the existence of acute mania or cerebritis, changes are determined by the great afflux of blood in both the cortical and medullary substance of the brain. The *grey or cortical substance* is separable in these cases into *three layers*, the inner, in contact with the white substance, preserves its grey appearance nearly unaltered; the outermost layer consists often of an evident white, thin, albuminous exudation, at other times of a pale colour. The essential changes occur in the middle layer placed between these two. It is of a bright red, from the excessive distension of its blood-vessels, and in the early stages of the affection of a firm consistence. When the delirium lessens in intensity, the colour becomes brownish, and the consistence of the pulp less firm. The *white substance*, being more compact, resists more the passage of the blood. Yet the injection of its capillaries gives its white colour a livid appearance, while ecchymoses, in the form of black spots, are found of varying extent. The fibrous appearance seen in the healthy brain is destroyed. If the inflammation be not arrested, the substance becomes soft and diffluent, a change which is promptly mortal. Suppuration or hæmorrhage may occur; and, if the disease become chronic, the white substance undergoes a slow induration.

c. *Acute Monomania.*—This may arise from the perversion of a single idea or one train of ideas, the reasoning powers continuing sound upon other subjects. This, the monomania of the intellect, is to be distinguished from the monomania of the propensities. This form of monomania usually arises from ambition or pride in men, and from vanity, love, or religion in women. They form some of the most irritable and intractable, of patients, persuaded, as they usually are, that their detention arises from machinations, and employing their reasoning powers in sustaining their chimeras. The distinction of the two forms of monomania above alluded to may, be illustrated by reference to *suicide*. Suicidal monomania, resulting from the perversion of the instinct of self-preservation, can seldom be prevented, and its victim seeks his destruction by any means that offers itself. The other form of suicidal monomania results from false perception and false judgment. A man believes himself detested by his family, or accused of dreadful crimes; another believes that in killing himself he is obeying God, and avoiding eternal damnation. Religious terrors easily overthrow weak intellects. These patients carefully conceal their design, will only terminate their lives by the description of death they have chosen, and often manifest a wonderful address and perseverance in accomplishing it.

D. *Cerebral Hallucinations.*—These are certain sensations which are roused in the brain of the insane by the mere excitement of that organ, without the actual presence of the objects which these sensations represent. Dreams are exact examples of hallucinations, for in them we see, hear, and touch objects which have no existence. The term illusion should be confined to affections of the senses and of sensibility. Hallucinations are the more worthy of study, because they usually precede the attack of mania, and, when they continue, can alone explain the tenacity of the erroneous ideas of the patient, and the secret motives of his determinations. As M. Archambaut observes, the man who hears a voice or sees a friend when no external object is near him; does not merely believe that he hears or sees this, but really does so: the sensation exists for him, although the senses are not actually excited. The frequent complication of insanity with hallucinations is difficult of remedy; for the patient judges and reasons from his hallucination as a reasonable man does from his sensations. In hallucination, all passes within the brain, the action of the senses forming no part of the impression: in illusions, perverted impressions of the external senses are conveyed to the cerebral centre.

2.—*In a State of Feebleness.*

A. *Chronic Delirium* (*Chronic Cerebritis*).—When acute mania is not cured it degenerates into chronic delirium, which is itself but the commencement, sooner or later, of a state of dementia. To the condition of excitement, the stage of debility has succeeded. The patient speaks little or none, and although his organic functions remain perfect, his intellect has lost its energy. Before degenerating into incurable dementia, during a lapse of time, sometimes a considerable one, this disease may remain nearly or quite stationary. It is difficult to decide during this period whether it is incurable or not, and a decided opinion must not be given until the expiration of two or three years, after which time a cure is rare. Short lucid intervals often deceive. So marked is the change of habits and ordinary ideas during the disease, that no hope of cure exists while this continues the case. The return to the ordinary tastes and sentiments alone presage it.

B. *Cerebral Œdema—Acute Dementia—Stupidity of Georget—Stupor of Orfila.*—Esquirol first described this disease. He observed that the insane are often seized with a rather sudden extinction of all their faculties, forming a very different condition to ordinary dementia, inasmuch as it almost always attacks young subjects, and terminates favourably. He termed it *acute dementia.* This affection, attended with temporary prostration of the intellcct, is dependent upon a particular œdematous condition of the cerebral conovlutions, quite distinct from the effusions known as acute or chronic hydrocephalus.

Upon examining cases which have proved fatal, the membranes of the brain are found thin, but much raised by the subjacent serum. The pia-mater is however thickened and much injected. The superior and lateral convolutions are flattened, and compressed against each other. The *grey substance,* in proportion to the quantity of the serum, loses its natural colour, and becomes spongy and infiltrated. Its vessels are gorged with the serum. Drops of this fluid ooze out as the part is cut. There is less apparent imbibition by the *white* portion, but drops of fluid may always be pressed out by the fingers. On tearing the white substance through, its capillaries, injected with serum, are visible. Of nine cases examined, one only presented fluid in all the cerebro-spinal cavities. In all the others the œdema was partial and circumscribed to the superior lateral, the anterior, or the posterior regions of the brain. In no case was the fluid found to have descended to the base of the brain. In one case only had it penetrated into the ventricles, which were ordinarily flattened and compressed. Cerebral œdema is not an isolated disease, and only developing itself accidentally in the insane ; it is part of a lymphatic predisposition, and accompanies usually œdema in other parts of the body, whether of the principal cavities, or the joints.

The symptoms of cerebral œdema are manifested first in a gradual lesion of the general *sensibility,* then of locomotion, and lastly of the intellect. As soon as the effusion commences, certain portions of the skin lose their sensibility, and in other cases become perverted as regards the perception of heat and cold. In many cases the insensibility of the skin is excessive, so that it may be pricked, burned, &c. without any pain; and the mucous membrane of the nostrils and the conjunctiva are similarly inirritable. As regards *locomotion,* the patient feels, at an early period, an indisposition and difficulty in moving his limbs. He is not paralysed, for, although the movements require an enormous effort, they are still possible. The patients will remain standing in one position for hours together. In some examples, a condition much resembling catalepsy was present. The lesion of the *understanding* is very marked. To the most violent agitation, or delirium, succeeds a perfect calm. The most extraordinary hallucinations torment the patient, his memory and powers of attention early become confused, his ideas vague, his articulation difficult or impossible. When the œdema is more advanced, complete stupor prevails, during which state of insensibility, however, a consciousness of his condition sometimes remains ; as those who have been cured have afterwards stated, although at the time they possessed neither the power or desire of expression of any kind. The alimentary canal suffers from a like atony; and hunger, thirst, and the excretions become impeded. The movements of the heart are remarkably lessened in number, the pulse falling sometimes to 40 or 30.

Unless accompanied by some grave complication, this disease, although of frequent occurrence among the insane, is rarely mortal; but is almost always cured after a duration of some months. In nine years the author has had but five opportunities of making an examination after death.

c. *Simple Dementia.*—This is the general enfeeblement of the moral and intellectual faculties which terminate all incurable deliriums; and is characterized by absence of all power of reasoning, forgetfulness of the past, and indifference as to the present and future. The organic functions may be completely executed, and the general health robust. As the affection advances it becomes complicated with paralysis, and for years before his death the patient may be confined to his bed. There generally exists in this condition a species of white induration of the cerebral substance. It presents different degrees in dementia and in complete idiotcy. In idiots, and especially in those in whom, as often happens, an entire hemisphere is atrophied, cerebral induration is very manifest, the texture of the brain being dense, elastic, and converted into an almost fibro-cartilaginous tissue. In dementia, the induration, although less considerable, is no less remarkable, whether as relates to its anatomical characters, or to its coincidence with the diminution of the cerebral functions. The brain, which in its natural state possesses a softish consistence and is traversed by such numbers of delicate vessels, becomes in dementia of a dull white colour, and of a hard, resisting, fibrous texture, in which the vessels have disappeared. The same induration is met with frequently in epilepsy, explaining the successive annihilation of the various intellectual faculties, which at last terminates in confirmed dementia.

3.—*In the State of Abolition,* (*Idiotcy and Imbecility.*)

Imbecility differs from idiotcy by not being congenital, but resulting, in individuals who have possessed their intellects, from a successive degradation slowly developed. Not only have idiots the intellectual organ malformed, but their whole economy participates usually in the condition of suffering. They are usually small, ill-developed, and short-lived. Others are rickety, scrofulous, epileptic, or paralysed. Almost all manifest the change in the cerebral structures already noticed. The indurated nervous tissue resembles an inorganic substance, which is best compared, as regards colour and density, to a hard egg. The cerebral substance is depressed, and the eye can detect no trace of capillary vessels. Submitted to the action of fire it becomes tough like horn, emits a strong and penetrating odour, and leaves a blackish, polished, compact residue. A portion of healthy brain similarly treated gives different results. It expands, emits scarcely any and leaves a light and brownish residue. The induration seems to affect especially the white rather than the grey substance. In the affected portions the nervous pulp may be torn into fibres, varying in direction, accordingly as the brain, the cerebellum, or the medulla, is the part in question, and corresponding with the most recent researches upon the structure of these parts. The elasticity and fibrous disposition of this species of induration sufficiently distinguish it from other forms occurring in other diseases of the nervous centres.

